# Inhibition of BRD4 prevents peribronchial fibrosis in mice with cutaneous lewisite exposure

**DOI:** 10.3389/fmolb.2025.1644792

**Published:** 2025-07-30

**Authors:** Huaxiu Zeng, Pooja Singh, Rajesh Sinha, Crystal T. Stephens, Aftab Ahmad, Mohammad Athar, Veena B. Antony

**Affiliations:** ^1^ Division of Pulmonary, Allergy, and Critical Care Medicine, Department of Medicine, University of Alabama at Birmingham, Birmingham, AL, United States; ^2^ Superfund Research Center, University of Alabama at Birmingham, Birmingham, AL, United States; ^3^ Department of Anesthesiology and Perioperative Medicine, University of Alabama at Birmingham, Birmingham, AL, United States; ^4^ Department of Dermatology, University of Alabama at Birmingham, Birmingham, AL, United States

**Keywords:** arsenical, BRD4 inhibitor, CPI-0610, lewisite, pulmonary fibrosis, fibrotic, inflammation

## Abstract

**Introduction:**

Arsenicals like lewisite are highly toxic vesicant chemical warfare agents that cause severe skin damage and systemic inflammation. Exposure activates cytokine release, leading to pulmonary injury, including edema, hemorrhage, and in severe cases, Bronchiolitis Obliterans Syndrome (BOS), marked by airway fibrosis and narrowing. The only approved treatment, British anti-lewisite (BAL), has limitations due to toxicity and field administration challenges. BRD4, a BET family protein, regulates inflammatory gene expression, and its inhibition has shown therapeutic potential. CPI-0610 (Pelabresib), a selective BRD4 inhibitor, is currently being explored for its anti-fibrotic and anti-inflammatory effects.

**Methods:**

In a murine model, we evaluated the therapeutic potential of CPI-0610 in mitigating lewisite-induced pulmonary damage. Mice were exposed to a single cutaneous dose of lewisite to induce systemic lung injury. Following exposure, one group of mice received CPI-0610 treatment, while a control group remained untreated. Lung tissues were harvested for molecular and histological analysis. The expression of inflammatory and fibrotic markers, including interleukin-6 (IL-6) and alpha-smooth muscle actin (α-SMA), was quantified via RT-PCR and immunohistochemistry.

**Results:**

Treatment with CPI-0610 significantly reduced the expression of IL-6 and α-SMA in lung tissues of lewisite-exposed mice compared to untreated controls. Histological analysis revealed reduced signs of inflammation, extracellular matrix deposition, and fibrotic remodeling in the CPI-0610 group. These findings indicate a protective effect of BRD4 inhibition on arsenical-induced lung injury.

**Discussion:**

Our study provides the first experimental evidence that BRD4 inhibition via CPI-0610 attenuates the development of pulmonary fibrosis following cutaneous lewisite exposure in mice. These results suggest that targeting BRD4 signaling can effectively reduce inflammation and fibrotic progression in the lungs. Given CPI-0610’s favorable clinical safety profile, it holds promise as a novel therapeutic strategy for treating arsenical-induced pulmonary complications, potentially improving outcomes where current countermeasures like BAL fall short. Further studies are warranted to explore its mechanism of action and therapeutic efficacy in broader exposure models.

## Introduction

The lung exhibits heightened susceptibility to arsenical toxicity compared to other organ systems, likely due to direct exposure routes and systemic absorption. Historical documentation and public testimonies from victims and veterans indicate that chronic pulmonary complications including fibrosis, chronic obstructive pulmonary disease (COPD), and carcinogenesis were observed in individuals exposed to lewisite and sulfur mustard (SM) during World War I (WWI) gas chamber incidents and postwar human subject studies (Amini et al., 2020; Ghabili et al., 2011). Epidemiological follow-ups and archival medical evaluations of exposed populations revealed persistent respiratory pathologies, such as bronchiolitis obliterans and reduced pulmonary function, decades after initial exposure (Ghanei et al., 2010). These effects are attributed to alkylating and oxidative damage mechanisms from SM, combined with arsenic-mediated mitochondrial dysfunction and inflammatory cascades triggered by lewisite (Jain et al., 2011). Despite anecdotal reports, systematic longitudinal data remain scarce due to ethical constraints and incomplete records of human experimentation during this period (Pechura and Rall, 1993). Such findings emphasize the long-term public health implications of chemical warfare agents on respiratory health.

Clinical manifestations of arsenical exposure frequently involve respiratory compromise, presenting as pharyngitis, persistent cough, and dyspnea (Parvez et al., 2013). Among arsenicals, lewisite a potent organic vesicant, induces rapid tissue injury characterized by severe pain, skin blistering, and systemic toxicity (Li, 2016a; King et al., 1992; Kehe et al., 2001; Lindsay et al., 2004; Sahu et al., 2013). Cutaneous exposure triggers immediate burning sensations, erythema within 30 min, and subsequent edema and bullae formation (Flora et al., 2009). Chronic effects may involve fibrotic remodeling of lung tissue, as observed in historical exposures (Nishimoto et al., 1970; Pechura and Rall, 1993). The acute phase of lewisite toxicity is marked by oxidative stress and inflammatory cascades, while delayed outcomes reflect persistent cellular damage and impaired repair mechanisms (Li et al., 2016b). These findings underscore the dual-phase pathology of cutaneous lewisite exposure, necessitating prompt decontamination, and targeted interventions to mitigate both immediate and long-term sequelae.

Organ fibrosis is a multifaceted pathological process initiated by external stimuli that activate fibroblasts and effector cells, leading to epigenetic alterations and fibrotic gene transcription. These changes trigger fibrotic signaling cascades, transforming cellular, tissue, and organ phenotypes. Epigenetic modifications, particularly histone acetylation, play a pivotal role in regulating fibrotic gene expression. Histone acetylation, catalyzed by histone acetyltransferases (HATs) and reversed by histone deacetylases (HDACs), disrupts chromatin compaction, enhancing accessibility for transcriptional machinery and promoting active gene transcription.

The Bromodomain and Extra-Terminal (BET) protein family, comprising BRD2, BRD3, BRD4, function as epigenetic readers by binding acetylated lysine residues on histones and non-histone proteins. BRD4, the most extensively studied BET protein, forms transcriptional complexes at genomic regions enriched with transcription factors and mediator complexes, playing a crucial role in various physiological and pathological processes, including fibrosis. Recent advances in the understanding of BRD4 have provided valuable insights into its role in fibrosis. Studies using the non-selective BET inhibitor JQ1 during the acute inflammatory phase have demonstrated reduced bleomycin-induced pulmonary fibrosis in mice, highlighting the significance of BET proteins in fibrosis development. However, the specific contributions of individual BET family members to myofibroblast transdifferentiation and Pulmonary fibrosis (PF) progression remain to be fully elucidated (Prinjha et al., 2012).

To explore potential therapeutic interventions, we evaluated CPI-0610, a BET inhibitor targeting BRD4, as a novel treatment strategy for arsenical-induced pulmonary fibrosis (PF). CPI-0610 treatment significantly reduced the intensity of opaque areas and hyperinflation on CT imaging, decreased airway resistance, and mitigated inflammatory cell infiltration around the airways in lewisite-exposed mice. Furthermore, molecular analyses confirmed that CPI-0610 effectively downregulated α-SMA expression, reduced extracellular matrix (ECM) deposition, and suppressed IL-6 levels. These findings suggest that CPI-0610 blocks PF in mice following cutaneous lewisite exposure and highlight its potential as a clinically relevant therapeutic option for arsenical-induced lung injury.

## Materials and methods

### Animal studies

All animal procedures were conducted after approval from the Institutional Animal Care and Use Committees of MRIGlobal and the University of Alabama at Birmingham (UAB). In the current study, adult male and female Ptch1 +/−/SKH-1 hairless mice (5–6 weeks) were obtained from Dr. Athar’s lab at UAB. Mice were housed in controlled environments (25°C, 12-h light/dark cycle) with *ad libitum* access to standard rodent chow and autoclaved water. All experiments adhered to the ARRIVE guidelines for reporting *in vivo* research, with acclimatization periods of ≥7 days prior to experimental interventions.

### Lewisite exposure procedures

All studies involving lewisite exposure were conducted using Ptch1+/−/SKH-1 mice at MRIGlobal (Kansas City, MO), as the handling, storage, and use of such hazardous chemicals in the United States are restricted to specialized facilities equipped for safe management and decontamination. Mice received topical lewisite application on a 2.56 cm^2^ dorsal skin area, following a standardized methodology for dermal chemical exposure (National Research Council (US) Subcommittee on Chronic Reference Doses for Selected Chemical Warfare Agents, 1999). Before lewisite exposure, mice were anesthetized by an Intraperitoneal (IP) injection of a mixture of 100 mg/kg of ketamine and 5–7 mg/kg of xylazine, and 0.05–0.1 mg/kg buprenorphine for pain relief. Anesthetized animals received a topical application of ethanol-diluted lewisite (30 μL) on a 2 cm^2^ area of dorsal skin, at a dose of 1.54 mg/kg. This dosage represents one-tenth of the LD50 and is comparable to a lewisite amount of approximately 32–40 μg, which is sufficient to induce vesication on human skin. At 24 h post-exposure, mice received additional analgesic support (0.05–0.1 mg/kg buprenorphine IP) and terminal anesthesia (150 mg/kg ketamine IP) prior to tissue collection, ensuring compliance with endpoints for minimizing distress (National Research Council (US) Subcommittee on Chronic Reference Doses for Selected Chemical Warfare Agents, 1999). All experiments were performed as per Institutional Animal Care and Use Committee (IACUC) guidelines and Environmental Health and Safety (EHS) regulations.

### CPI-0610 intraperitoneal (IP) injection

CPI-0610 (pelabresib) was administered via intraperitoneal (IP) injection at a dose of 10 mg/kg body weight, prepared in a vehicle solution of dimethyl sulfoxide (DMSO) and methyl cellulose to ensure solubility and biocompatibility, as standardized in preclinical BET inhibitor studies (Blum et al., 2022; Verstovsek et al., 2021). The dosing regimen (twice weekly) and concentration align with established posology for BET inhibitors in murine models, despite differences in clinical administration routes (intramuscular or oral). Fresh CPI-0610 solutions were prepared under sterile conditions prior to each injection to maintain stability and efficacy, adhering to guidelines for experimental compound handling (National Research Council (US) Subcommittee on Chronic Reference Doses for Selected Chemical Warfare Agents, 1999). This IP delivery protocol was selected to optimize systemic bioavailability and replicate pharmacodynamic profiles observed in prior BET inhibitor trials (Mirguet et al., 2013; Albrecht et al., 2016).

### Micro-CT scan

Micro-computed tomography (micro-CT) imaging was performed using a small-animal Single Photon Emission Computed Tomography (SPECT) system, specifically the X-SPECT (Gamma Medica, Inc.), integrated with a CT scanner. The CT imaging parameters were optimized for high-resolution visualization of murine lung architecture, with a spatial resolution of 150 μm and a field of view of 79 mm, allowing for detailed examination of the entire thoracic cavity. Image processing and analysis were conducted using custom software developed in LabVIEW v17.0 (National Instruments, Austin, TX, United States).

### Lung function test

Lung function tests were performed using a FlexiVent apparatus (SCIREQ, Montreal, Canada) to assess airway resistance in anesthetized mice. The ventilation parameters were set at 160 breaths/min, with a tidal volume of 6 mL/kg and a positive end-expiratory pressure of 2.5 cm H2O, as optimized for murine respiratory mechanics. Mice were anesthetized using a ketamine/xylazine mixture administered intraperitoneally, following which a tracheostomy was performed to facilitate direct airway access. Airway hyperresponsiveness was evaluated by exposing the animals to serially increasing concentrations of aerosolized methacholine (0, 10, 20, 30, and 50 mg/mL), a bronchoconstrictive agent commonly used to assess airway reactivity. Respiratory system resistance was measured in response to each methacholine dose, providing a comprehensive profile of airway responsiveness. Data acquisition and analysis were conducted using the FlexiVent system’s proprietary software, with results expressed as total pulmonary system resistance. This protocol allows for the quantitative assessment of airway function and reactivity, crucial for evaluating the effects of experimental interventions or disease models on respiratory mechanics (Blum et al., 2022).

### Hydroxyproline enzyme-linked immunosorbent assay (ELISA)

Mice lungs were removed, weighed, and homogenized. Collagen content was assessed by Hydroxyproline Assay Kit (Colorimetric) (Abcam, ab222941) following the manufacturer’s instructions. Briefly, samples and standards were incubated with chloramine-T and dimethylaminobenzaldehyde (DMAB) to form a chromogenic complex, with absorbance read at 560 nm. Collagen content was calculated using a hydroxyproline-to-collagen conversion factor of 1:10. Data was normalized to lung wet weight (μg collagen/mg tissue) and expressed as fold change relative to controls. Assays included triplicate technical replicates, and quality controls (hydroxyproline standards, reagent blanks) were incorporated to ensure precision (Li et al., 2016c). This method provides a reliable biochemical correlation of fibrosis severity in experimental models of lung injury.

### Western blotting

Protein expression analysis was performed via Western blotting to evaluate molecular markers in murine lung tissues. Lungs were homogenized in RIPA buffer supplemented with protease inhibitors (Thermo Fisher Scientific), and protein concentrations were quantified using a BCA assay. Proteins (20–30 µg/lane) were resolved on 4%–20% gradient polyacrylamide gels (Bio-Rad, Hercules, CA) under reducing conditions and transferred to PVDF membranes (MilliporeSigma, MA) using a semi-dry transfer system. Membranes were blocked with 5% non-fat milk in Tris-buffered saline containing 0.1% Tween-20 (TBST) for 1 h at room temperature and incubated overnight at 4°C with the following primary antibodies: anti-BRD4 (1:1000; Abcam ab245285), anti-α-SMA (1:2000; Cell Signaling Technology D4K9N), anti-IL-6 (1:1000; Abcam ab238132), anti-H3K9ac (1:2000; ThermoFisher MA5-11195), anti-H3 (1:2000; Abcam ab18521), and anti-β-actin (1:5000; Santa Cruz Biotechnology sc47778) as a loading control. After TBST washes, membranes were incubated with HRP-conjugated anti-rabbit or anti-mouse secondary antibodies (1:10,000; MilliporeSigma AP187P, AP130P) for 1 h at room temperature. Protein bands were visualized using enhanced chemiluminescence (ECL) substrate on an ImageQuant 800 imaging system (Cytiva). Band intensities were quantified using ImageJ software (NIH), normalized to β-actin or histone H3, and expressed as fold changes relative to control groups.

### Hematoxylin and eosin (H&E) staining

Lung tissues were fixed in 10% neutral buffered formalin for 24–48 h to preserve histoarchitecture, followed by paraffin embedding using standard protocols. Paraffin blocks were sectioned at 5 μm thickness and mounted onto glass slides. Sections were deparaffinized in xylene (2 × 5 min) and rehydrated through a graded ethanol series (100%, 95%, 70%) to distilled water. H&E staining was performed by immersing slides in hematoxylin (3–5 min) for nuclear staining, rinsing in tap water, differentiating in acid alcohol (1% HCl in 70% ethanol), and counterstaining with eosin (1 min) for cytoplasmic and extracellular matrix visualization. Slides were dehydrated in ethanol, cleared in xylene, and coverslip applied with a resinous mounting medium. Three independent tissue sections per experimental group were analyzed to ensure representative sampling. Brightfield imaging was conducted using a Keyance BZ-X800 microscope (Keyance Corporation, Osaka, Japan) equipped with 10×–40× objectives.

### Immunohistochemistry (IHC)

Lung tissues were fixed in 10% neutral buffered formalin for 24–48 h, paraffin-embedded, and sectioned into 7 μm-thick slices. Sections were deparaffinized in xylene, rehydrated through graded ethanol, and subjected to antigen retrieval using citrate buffer (pH 6.0, 95°C, 20 min) to unmask epitopes. Endogenous peroxidase activity was quenched with 3% H_2_O_2_ followed by blocking in 5% normal goat serum to reduce nonspecific binding. Slides were incubated overnight at 4°C with primary antibodies: anti-BRD4 (1:200, Abcam ab245285) and anti-α-smooth muscle actin (α-SMA) (1:200, Cell Signaling Technology D4K9N). After washing, slides were treated with HRP-conjugated secondary antibodies and developed with 3,3′-diaminobenzidine (DAB) to visualize antibody-antigen complexes. Nuclei were counterstained with hematoxylin, and slides were dehydrated, cleared in xylene, and coverslipped. For analysis, three representative sections per group were imaged at ×200 magnification using a Keyance BZ-X800 microscope (Keyance Corporation). BioQuant Image Analysis Software (BioQuant, Nashville, TN) quantified staining intensity by measuring optical density (OD) of DAB signals in five random fields per section, normalized to hematoxylin-counterstained nuclei. α-SMA-positive areas (%) and BRD4 nuclear expression were calculated to assess myofibroblast activation and epigenetic regulation, respectively.

### Epithelial thickness

Slides were stained for picrosirius, and morphometric analysis was performed. Using ImageJ software, five airways per section, each with a diameter of 150–300 μm, were randomly selected for analysis. Epithelial thickness was measured and expressed as square micrometers per micrometer of basement membrane (BM) length.

### Reverse transcription-polymerase chain reaction (RT-PCR)

RNA was isolated from mice lung tissue by using RNeasy Plus Mini Kit (QIAGEN) according to manufacturer’s isolation protocol. RNA was reverse transcribed using iScript™ Reverse Transcription Supermix (Bio-Rad, Hercules, CA) in a GeneAmp PCR System 9700 (Applied Biosystems) according to manufacturer’s instructions. cDNA was amplified using Powerup™ SYBR™ Green Master Mix (Applied Biosystems, Foster City, CA) and gene-specific SYBER™ primers and probes against BRD4 in a Stepone Plus Real-time PCR System (Applied Biosystems, Foster City, CA), as well as the housekeeping gene. The reaction mixtures were subjected to 1 cycle of 2 m at 95°C; 39 cycles of 30 s at 95°C, 30 s at 56.4°C, 1 min at 72°; 1 cycles of 30 s at 95°C, 30s at 56.4°C, 10 min at 72°C. The following SYBR Green primer sequences were used: BRD4 forward: AGGCAAAAGGAAGAGGACGA, BRD4 reverse: CCGGTGAAGGAGTCTGAAGT; α-SMA forward: CCCAACTGGGACCACATGG α-SMA reverse: TACATGCGGGGGACATTGAAG; Gapdh forward: GGGTCCCAGCTTAGGTTCAT, Gapdh reverse: GTGTGCAGTTCCGGCTCTTAC. Prior to experimental use, standard curves and primer efficiencies were determined for the primer pairs above. Following all experimental RT-qPCR, relative changes in gene expression were quantified compared to control housekeeping gene using the ΔΔCt method.

### Statistical analysis

Data is expressed as the mean ± SEM. Statistical analyses were carried out using GraphPad Prism 10.0. One-/Two-way ANOVA was used to compare the differences between groups. After the ANOVA analysis, the *post hoc* multiple comparisons were performed by using Tukey’s honestly significant difference test to determine the statistical difference from each other among subgroups unless otherwise indicated. P < 0.05 was considered significant.

## Results

### CPI-0610 ameliorates lewisite-induced bronchiolitis obliterans

**FIGURE 1:**
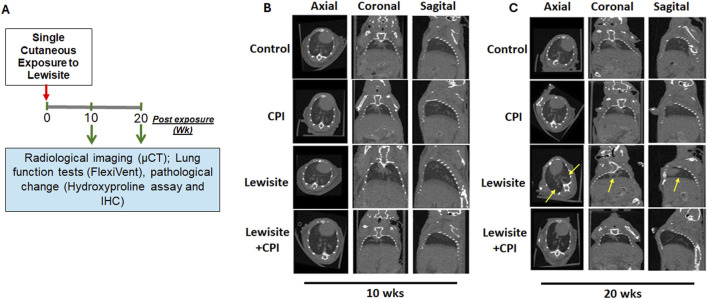
CPI-0610 causes long-term effects on Lewisite-damaged lungs. **(A)** A single dose of lewisite (1.54 mg/kg) was applied to the skin of mice. Flowchart shows the time points of the experiment and potential readouts. **(B)** Representative micro-computed tomography (micro-CT) imaging axial, coronal, and sagittal projections of lungs in control, CPI-0610, lewisite, and lewisite + CPI-0610 mice groups at 10 weeks. **(C)** Representative micro-CT imaging axial, coronal, and sagittal projections of lungs in control, CPI-0610, lewisite and lewisite + CPI-0610 mice groups at 20 weeks. n = 5-6, each group.

Micro-CT analysis was employed to assess lewisite-induced chronic lung injury and the therapeutic efficacy of CPI-0610 across four experimental groups: control, CPI-0610 alone, lewisite exposure, and lewisite + CPI-0610 treatment (Figure 1A). At 10 weeks post-single cutaneous lewisite exposure, lungs exhibited hallmarks of PF, characterized by increased opacities and hyperinflation compared to control tissues. Notably, intraperitoneal administration of CPI-0610 attenuated these pathological features, evidenced by reduced intensity of opaque areas and diminished hyperinflation, suggesting a decrease in inflammation and fibrosis relative to the lewisite-exposed group. Extended analysis at 20 weeks post-exposure revealed a dynamic progression of lung pathology in the lewisite group, with a reduction in opaque area intensity but an increase in consolidated regions and hyper expansion compared to controls. CPI-0610 treatment consistently mitigated these long-term effects, demonstrating decreased peribronchiolar opacification and hyperinflation compared to untreated lewisite-exposed lungs (Figures 1B,C). Importantly, CPI-0610 was well-tolerated at the administered dose, aligning with its current evaluation in early-stage human clinical trials for other indications. These findings suggest that CPI-0610, a BET inhibitor, may offer therapeutic potential in preventing reversing chronic lung dysfunction induced by cutaneous lewisite exposure, warranting further investigation into its mechanisms of action and long-term efficacy in chemical-induced lung injury models.

### CPI-0610 mitigates lewisite-induced lung function impairment

**FIGURE 2:**
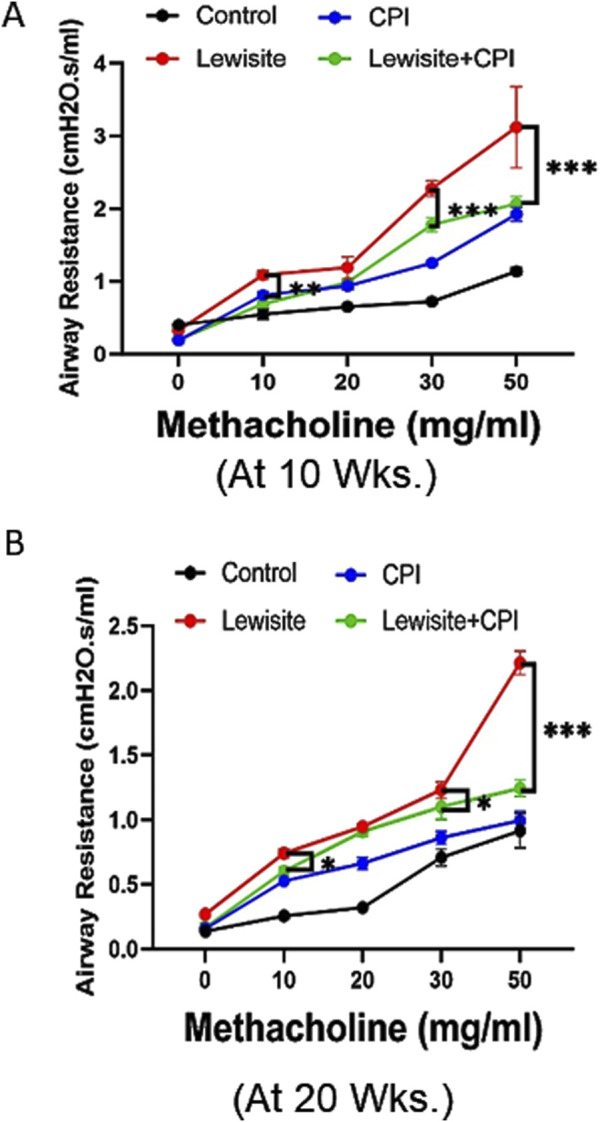
CPI-0610 decreases airway resistance in mice. Respiratory mechanics analysis of lewisite exposed mice at 10 and 20 weeks. **(A)** Airway hyperreactivity assessments at different concentrations of methacholine in control, CPI-0610, lewisite, and lewisite + CPI-0610 mouse groups at 10 weeks. **(B)** Airway hyperreactivity assessments at different concentrations of methacholine in control, CPI-0610, lewisite, and lewisite + CPI-0610 mouse groups at 20 weeks. Each dot represents an individual mouse. **(A,B)**, *P < 0.05 and ***P < 0.001 versus controls and **P < 0.01, using two-way ANOVA. n = 5–6, each group.

Lung function assessment using FlexiVent revealed significant functional deterioration in mice 10 weeks after cutaneous lewisite exposure (Figure 2A). The lung compliance examination demonstrated abnormal lung function, indicative of the chronic effects of lewisite on pulmonary mechanics. Treatment with CPI-0610 showed a protective effect against lewisite-induced airway hyperresponsiveness, as evidenced by attenuated increases in airway resistance in response to higher doses of methacholine challenge. This therapeutic benefit persisted at 20 weeks post-exposure (Figure 2B), suggesting long-term efficacy of CPI-0610 in preserving pulmonary function. Quantitatively, the difference in lung compliance between CPI-0610-treated and untreated lewisite-exposed mice showed a modest change from 1.05 cmH2O.s/mL to 0.97 cmH2O.s/mL at the highest methacholine dose (50 mg/mL) when comparing 10-week and 20-week timepoints. This sustained improvement in lung compliance indicates that CPI-0610 effectively counteracts the lewisite-induced decline in lung mechanics. Collectively, these data demonstrate that CPI-0610 treatment significantly inhibits the compromised lung function associated with cutaneous lewisite exposure, potentially by modulating the underlying inflammatory and fibrotic processes that contribute to airway remodeling and reduced lung elasticity in chemical-induced pulmonary injury.

### CPI-0610 therapy blocks pathological features of lewisite-induced airway fibrosis

**FIGURE 3:**
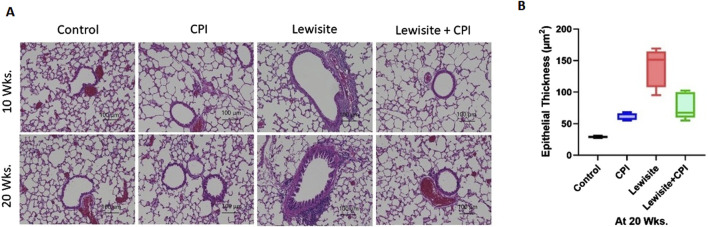
CPI-0610 attenuates bronchiolitis obliternas in mice. **(A)** Histopathological assessment of lung sections stained with hematoxylin and eosin at Weeks 10 and 20. Scale bars, 100 mm, showing airway remodeling in lewisite and lewisite + CPI-0610 mouse groups at 10 and 20 weeks. Scale bars, 100 μm. **(B)** Measurements of epithelial thickness of airways at 20 weeks. The line within the box represents the median. ***P < 0.001 and ****P < 0.0001 versus Lewisite; P values were determined using one-way ANOVA with Bonferroni *post hoc* method. n = 5–6, each group.

Histopathological analysis using H&E staining revealed significant lung damage in mice 10 weeks after cutaneous lewisite exposure (Figure 3A). Key pathological features included airway narrowing, disruption of alveolar structure, and inflammatory cell infiltration. Pulmonary peribronchial fibrosis (PF) was evidenced by scarring and thickening of tissue surrounding and between air sacs. The progression of PF was observed at 20 weeks, characterized by mature eschar formation and increased neutrophil infiltration, indicating a worsening of fibrotic changes over time in lewisite-exposed mice. These observations collectively confirm the development of PF in the cutaneous lewisite exposure model. Notably, treatment with CPI-0610 significantly attenuated lewisite-induced inflammatory cell infiltration at both 10- and 20-week time points. Further analysis at 20 weeks focused on airway epithelium thickness, a key indicator of fibrotic remodeling. The airway epithelium height was markedly increased in the lewisite exposure group compared to controls, while the lewisite + CPI-0610 group showed a significant reduction in epithelial thickness relative to the untreated lewisite group (Figure 3B). These findings demonstrate that CPI-0610 therapy effectively prevents multiple pathological features associated with lewisite-induced pulmonary fibrosis, including inflammatory infiltration and airway remodeling, suggesting its potential as a therapeutic intervention for chemical-induced lung injury.

### CPI-0610 therapy attenuates pathological features of lewisite-induced peribronchial fibrosis

**FIGURE 4:**
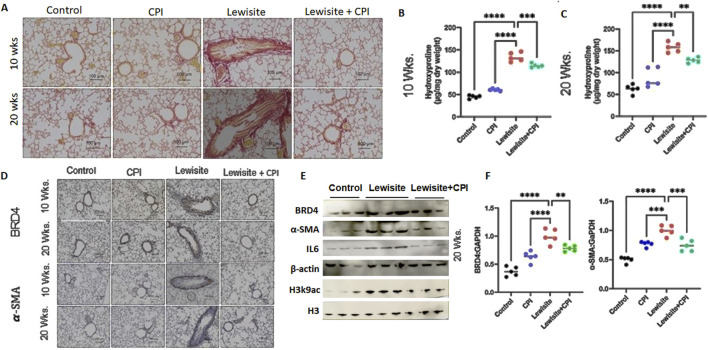
Cutaneous exposure to lewisite/DPCA causes airway remodeling in mice. **(A)** Representative lung histology with Picrosirius staining showing collagen deposition around airways at Weeks 10 and 20. Scale bars, 100 mm. **(B)** Hydroxyproline content at 10 weeks. **(C)** Hydroxyproline content for 20 weeks. **(D)** Representative lung histology with *α* -SMA (alpha smooth muscle actin) and BRD4 staining at Weeks 10 and 20. Scale bars, 100-mm. **(E)** Immunoblot analysis in whole lung tissue lysates for BRD4, *α* -SMA, and IL-6. β-actin expression was used as loading control. **(F)** Analysis of lung BRD4 and *α* -SMA mRNA by real-time PCR. *P < 0.05, **P < 0.01, and ***P < 0.001 using one-way ANOVA with Bonferroni *post hoc* method. n = 5–6, each group.

Picrosirius red staining under polarized light microscopy revealed increased collagen deposition in the airway walls of lewisite-exposed mice at both 10 and 20 weeks, with more pronounced accumulation at the later timepoint (Figure 4A). CPI-0610 treatment significantly reduced this collagen deposition at both timepoints. Quantitative analysis of hydroxyproline content corroborated these findings, showing elevated levels in lewisite-exposed lungs at 10 weeks, which were attenuated by CPI-0610 treatment (Figure 4B). At 20 weeks, collagen accumulation was further increased in lewisite-exposed lungs, but CPI-0610 treatment led to a significant reduction (p < 0.01) in collagen deposition (Figure 4C). Immunohistochemistry for α-SMA, a marker of myofibroblasts and tissue fibrogenesis, demonstrated fibrotic and hypertrophic areas around airways at 10 weeks post-lewisite exposure, accompanied by upregulation of BRD4 (Figure 4D). CPI-0610 treatment reduced both α-SMA and BRD4 expression. At 20 weeks, α-SMA-positive fibrotic scars obstructed small airways in lewisite-exposed lungs, but CPI-0610 treatment significantly reduced this hyperresponsiveness. Western blot and qPCR analyses at 20 weeks showed more pronounced improvements in α-SMA and BRD4 protein (Figure 4E) and mRNA levels (Figure 4F; p < 0.01) compared to 10 weeks. Lewisite exposure significantly increased α-SMA, BRD4, IL-6, and H3K9ac levels relative to controls, while CPI-0610 treatment effectively reduced these markers. These results indicate that CPI-0610 reverses lewisite-induced pulmonary fibrosis by modulating key fibrotic mediators and epigenetic regulators. In conclusion, the reduction in α-SMA expression, ECM deposition, and IL-6 levels in CPI-0610-treated lungs demonstrates a convincing ability to reverse pulmonary fibrosis associated with cutaneous lewisite exposure, highlighting the therapeutic potential of BET inhibition in chemical-induced lung injury.

## Discussion

Bronchiolitis Obliterans Syndrome (BOS), also known as obliterative or constrictive bronchiolitis, is a rare and serious lung disease characterized by inflammation and fibrosis leading to obstruction of the smallest airways (bronchioles). Specifically, in the context of toxic exposures (such as arsenicals like lewisite), injury to the airway epithelium and surrounding structures triggers an inflammatory response followed by subepithelial and peribronchiolar collagen deposition. This process is responsible for the constrictive remodeling and fibrotic thickening of the bronchiolar walls. Over time, this fibrosis compresses the airway, causing the classic features of constrictive bronchiolitis. BOS symptoms typically include a persistent dry cough, shortness of breath, wheezing, and fatigue, often worsening over weeks to months. Diagnosis relies on clinical history, pulmonary function tests showing airflow obstruction, and imaging, as chest X-rays may appear normal. While there is currently no cure and the disease is irreversible, treatments like corticosteroids or immunosuppressive agents may slow progression, though outcomes are often poor. In the context of environmental or occupational exposure, such as to lewisite or other toxic chemicals, BOS may present with insidious onset and progressive respiratory decline, highlighting the importance of recognizing and mitigating hazardous exposures.

In this study, we identify the BRD4 chromatin reader protein as a critical regulator of PF following single cutaneous exposure to lewisite. BRD4, a pro-fibrotic member of the BET family, functions autonomously in fibroblasts to drive fibrotic remodeling of the airway. Previous studies have implicated BRD4 as a key target in fibrotic gene networks, including those involved in lung fibrosis (Tang et al., 2013a; Tang et al., 2013b). Our integrative approach combined micro-CT imaging with pulmonary function assessments to identify structural and functional biomarkers of lung pathology in a murine fibrosis model. Micro-CT analysis (Figure 1) quantified regional ventilation changes and revealed reduced lung opacities alongside hyperinflation following CPI-0610 treatment, while spirometric measurements demonstrated dose-dependent improvements in lung compliance across methacholine challenge levels (Figure 2). In disease progression, micro-CT detected compensatory lung volume expansion and heterogeneous ventilation deficits, paralleling functional deteriorations in airway resistance and compliance. Histopathological validation through IHC and picrosirius red staining correlated these imaging biomarkers with molecular evidence of BRD4-mediated fibrotic gene regulation and collagen deposition, supported by hydroxyproline quantification (Figures 3, 4). Pathological examinations confirmed structural remodeling features including epithelial hyperplasia, inflammatory infiltration, and airway wall thickening, consistent with micro-CT-derived tissue density metrics. This multimodal platform enables longitudinal tracking of dynamic disease processes and therapy responses through quantitative structure-function correlations. This demonstrates that BRD4 is a central driver of PF development in lewisite-exposed mice. Gene expression analyses revealed a correlation between BRD4 protein abundance and mRNA expression in lewisite-induced PF (Figure 4F). This association aligns with BRD4’s role in stimulating transcription through interactions with positive transcription elongation factor-b (pTEFb) and RNA polymerase II (RNA Pol II) (Kulikowski et al., 2021). The upregulation of H3K9ac observed in this study likely facilitates genome-wide redistribution of BRD4 to activate pro-fibrotic genes following lewisite exposure (Figures 4E,F). These findings highlight the epigenetic mechanisms by which BRD4 drives PF. Currently, the only FDA-approved arsenical antidote, British Anti-Lewisite (BAL), has significant limitations (Mückter et al., 1997; Hughes, 1946), including systemic toxicity and the need for intramuscular administration. In contrast, our study demonstrates that CPI-0610, a less toxic and more effective BET inhibitor, reverses lewisite-induced PF. CPI-0610 acts by competitively displacing BET proteins from acetyl-histones on active enhancers, disrupting downstream signaling to RNA Pol II and reducing H3K9ac levels (Filippakopoulos et al., 2010). This mechanism effectively suppresses BRD4-driven fibrotic pathways.

CPI-0610 (pelabresib) is an investigational, orally bioavailable, small-molecule inhibitor of Bromodomain and Extra-Terminal (BET) proteins, with specific activity against BRD4 (Blum et al., 2022; Verstovsek et al., 2021; Yamatani et al., 2022; Gomes and Harrison, 2023). This compound effectively targets bromodomains, disrupting BET protein-mediated transcriptional activation of key genes involved in oncogenesis and inflammation (Dhalluin et al., 1999; Filippakopoulos et al., 2010; Belkina et al., 2013; Hammitzsch et al., 2015; Mele et al., 2013; Cheung et al., 2017). CPI-0610 demonstrates superior pharmacological properties compared to JQ1, an earlier BRD4 inhibitor (Mirguet et al., 2013) and is currently undergoing clinical development with a manageable safety profile for hematologic malignancies, particularly myelofibrosis (Albrecht et al., 2016; Sun et al., 2020; Stein et al., 2024; Mascarenhas et al., 2023). The most recent application of CPI-0610 is in the MANIFEST-2 study, a placebo-controlled trial evaluating the efficacy and safety of CPI-0610 (pelabresib) in combination with ruxolitinib versus placebo plus ruxolitinib in JAK inhibitor-naïve myelofibrosis patients (Harrison et al., 2022; Gomes and Harrison, 2023). Despite these promising developments in hematological malignancies, the potential therapeutic effects of CPI-0610 on cutaneous arsenical-induced chronic lung injury remain unexplored. This gap in research presents an opportunity to investigate the broader applications of BET inhibition in chemical-induced tissue damage and chronic inflammatory conditions. Previous studies have demonstrated the development of chronic lung injury, specifically constrictive bronchiolitis, in mice following a single cutaneous exposure to arsenicals (Surolia et al., 2023). In the current study, we investigated the role of BRD4 dysregulation in the progression of obstructive pulmonary injury after a single cutaneous exposure to lewisite in mice.

Additionally, we show that CPI-0610 rescues lewisite-induced PF in mice. Histological analyses at 10 and 20 weeks post-lewisite exposure showed decreased α-SMA expression and ECM deposition in CPI-0610-treated lungs. These findings suggest that CPI-0610 suppresses fibroblast activation and ECM accumulation by modulating α-SMA and collagen expression. Furthermore, CPI-0610 reduced inflammatory cytokine IL-6 expression, likely through downregulation of BRD4 and its H3 histone acetylation marks (Figures 4E,F). Collectively, these results demonstrate that CPI-0610 effectively mitigates the pro-fibrotic response, ECM deposition, and inflammation associated with lewisite-induced PF.

In conclusion, our study provides the first experimental evidence that pharmacological inhibition of BRD4 with CPI-0610 prevents the development of arsenical-induced PF in a murine model. By targeting pro-fibrotic gene expressions, ECM depositions, and inflammatory cytokines such as IL-6, CPI-0610 offers a novel therapeutic approach for chemical-induced lung injury. These findings pave the way for future studies exploring the potential of CPI-0610 as a treatment for arsenic-induced chronic lung injury due to its manageable safety profile and robust anti-fibrotic effects.

## Data Availability

The original contributions presented in the study are included in the article/Supplementary Material, further inquiries can be directed to the corresponding author.
