# Supplementary figures and images for: Inhibition of BRD4 prevents peribronchial fibrosis in mice with cutaneous lewisite exposure
Source: Front Mol Biosci. 2025 Jul 30;12:1644792. doi: 10.3389/fmolb.2025.1644792 (PMC12343216; doi:10.3389/fmolb.2025.1644792)

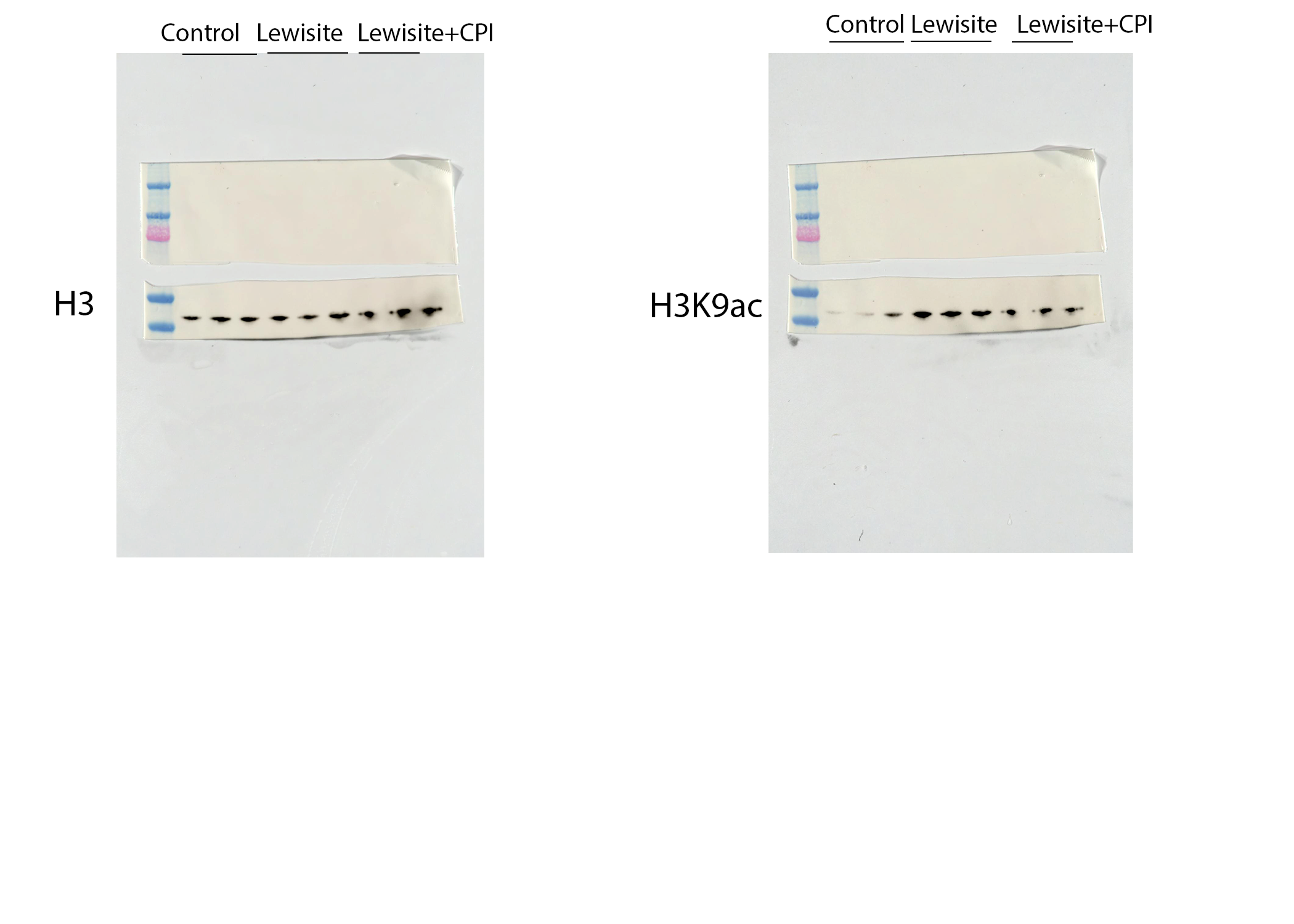

Supplement: Supplementary file 1 [file Image3.tif]

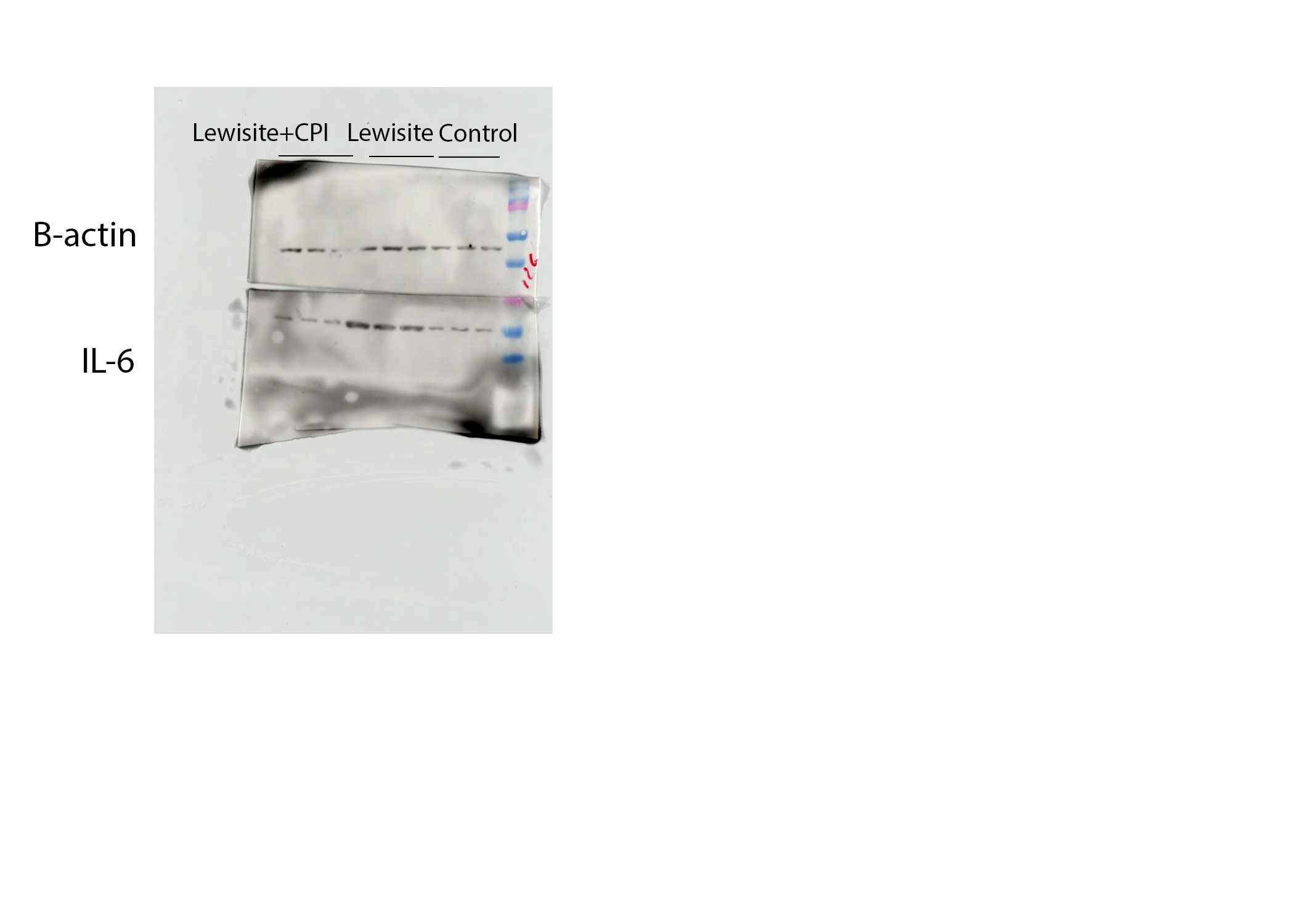

Supplement: Supplementary file 2 [file Image2.tif]

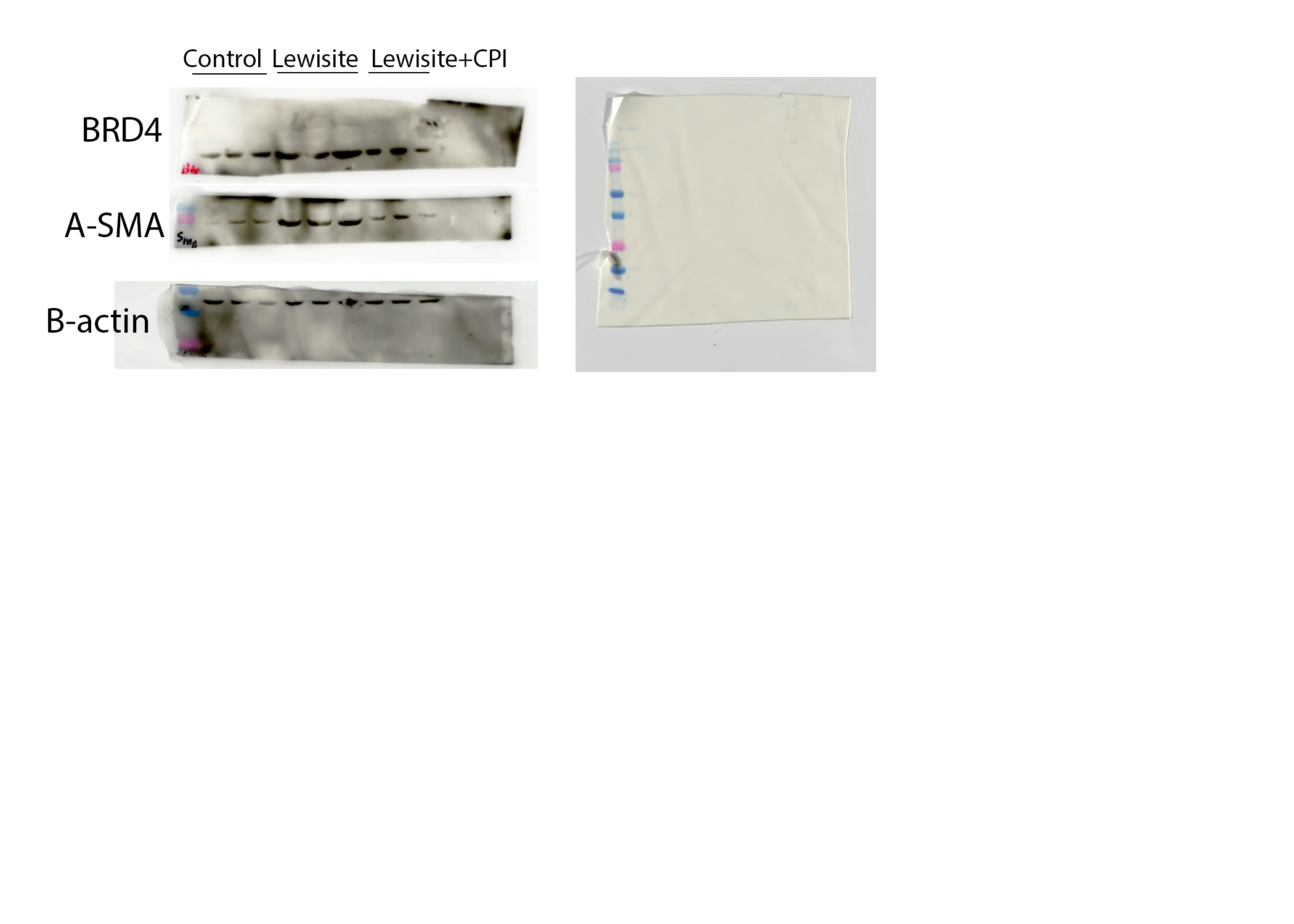

Supplement: Supplementary file 3 [file Image1.tif]
